# Dysregulated Iron Homeostasis as Common Disease Etiology and Promising Therapeutic Target

**DOI:** 10.3390/antiox12030671

**Published:** 2023-03-09

**Authors:** Bruce E. Holbein, Christian Lehmann

**Affiliations:** 1 Department of Microbiology and Immunology, Dalhousie University, Halifax, NS B3H 1X5, Canada; 2 Department of Anesthesia, Pain Management and Perioperative Medicine, Dalhousie University, Halifax, NS B3H 1X5, Canada

**Keywords:** iron homeostasis, iron dysregulation, inflammation, infection, cancer, iron chelation

## Abstract

Iron is irreplaceably required for animal and human cells as it provides the activity center for a wide variety of essential enzymes needed for energy production, nucleic acid synthesis, carbon metabolism and cellular defense. However, iron is toxic when present in excess and its uptake and storage must, therefore, be tightly regulated to avoid damage. A growing body of evidence indicates that iron dysregulation leading to excess quantities of free reactive iron is responsible for a wide range of otherwise discrete diseases. Iron excess can promote proliferative diseases such as infections and cancer by supplying iron to pathogens or cancer cells. Toxicity from reactive iron plays roles in the pathogenesis of various metabolic, neurological and inflammatory diseases. Interestingly, a common underlying aspect of these conditions is availability of excess reactive iron. This underpinning aspect provides a potential new therapeutic avenue. Existing hematologically used iron chelators to take up excess iron have shown serious limitations for use but new purpose-designed chelators in development show promise for suppressing microbial pathogen and cancer cell growth, and also for relieving iron-induced toxicity in neurological and other diseases. Hepcidin and hepcidin agonists are also showing promise for relieving iron dysregulation. Harnessing iron-driven reactive oxygen species (ROS) generation with ferroptosis has shown promise for selective destruction of cancer cells. We review biological iron requirements, iron regulation and the nature of iron dysregulation in various diseases. Current results pertaining to potential new therapies are also reviewed.

## 1. Introduction

When oxygen first appeared in the Earth’s atmosphere, iron with its Fe^2^/Fe^3^ redox potential of 0.771 V represented a sweet spot for chemical catalysis of a wide range of oxidation and reduction reactions needed for biosynthesis and energy production by all living organisms, except for a few microbes. Around 2% of human genes encode for iron proteins, with more than half of these having catalytic function, with oxidoreductase class enzymes being the largest fraction [1].

### 1.1. Iron as Biological Catalyst

Table 1 provides examples of key iron-dependent enzymes needed by most cells for critical functions at a number of physiological levels. These enzymes are critically important for cell growth and replication and generally, iron cannot be substituted for activity in these. This underlies the irreplaceable need for iron by all higher organisms.

### 1.2. Excess Iron Toxicity

**Scheme 1:**
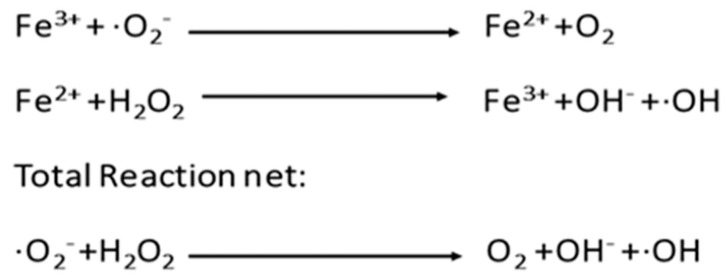
Reactive oxygen species generation.

Seemingly paradoxical, iron is also toxic to cells because of its production of oxidizing radicals with their formation driven by redox cycling of iron. Superoxide (O_2_^−^) is produced constitutively by the electron transport chain in cells by slippage of an electron to O_2_ with concomitant reduction of Fe^3^ to Fe^2^. The human body produces around 5 g O_2_^−^/day [9], but superoxide dismutase (an iron dependent enzyme) normally neutralizes quantities in excess of normal amounts as needed for cell regulation and signaling [10]. Superoxide is also produced by nicotinamide adenine phosphate oxidase in the phagocytic defensive cells of vertebrate animals and this is used to kill phagocytosed invading microbes [11]. Superoxide can set up a cascade cycling of Fe reduction/oxidation coupled to peroxide and hydroxyl radical ·OH production, as shown in Scheme 1, with ·OH being highly toxic through damage to DNA and membrane lipids [12].

### 1.3. Chemical Containment

Containment of reactive iron and its ROS products is achieved through both compartmentalization and its chemical chaperoning. Phagocytes, including macrophages and polymorphonuclear leukocytes, compartmentalize ROS production within intracellular phagosomes, which spares other intracellular organelles and the extracellular milieu from direct ROS exposure [13]. The bulk of total body iron stores represents around 55 mg/kg and is located intracellularly, safely incorporated within hemoglobin (erythrocytes), myoglobin (muscle cells) and ferritin (liver cells) [14]. Iron incorporated into heme and ferritin is not freely chemically available to participate in ROS production. Body iron stores are shuttled to sites of use in other cells by transferrin, which binds iron avidly so that it is effectively chaperoned in circulation and not available for ROS reactions. There is an additional small labile iron pool located both intracellularly and extracellularly which is readily ROS-reactive [15], and this pool is important in relation to iron dysregulation, which increases the amounts in this labile pool.

It is interesting to note that some currently used therapeutic agents, including aminoglycoside antibiotics, can bind iron not fully satisfying its chaperoning requirements [16] and can induce ROS-related tissue damage [17], likely due to the mobilization of labile iron. This underscores the need for containment and chaperoning of labile ROS-reactive iron in the host as part of normal homeostatic control.

## 2. Regulation of Normal Iron Homeostasis

**Figure 1:**
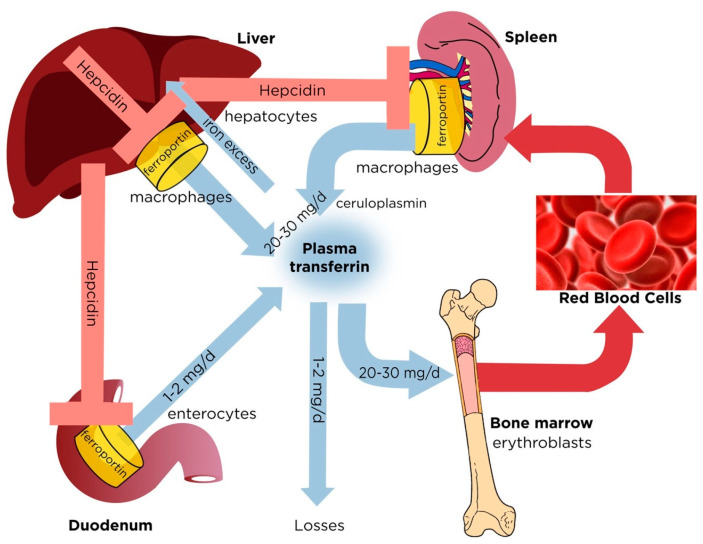
Dynamics of systemic iron balance. Plasma transferrin delivers iron to bone marrow erythroblasts and to other tissues. It contains a very small (∼0.1%) but highly dynamic fraction of body iron that turns over >10 times/day to meet the iron need for erythropoiesis (20–30 mg/day). The transferrin iron pool is primarily replenished with iron recycled from hepatic and splenic macrophages during erythrophagocytosis of senescent red blood cells. Duodenal enterocytes absorb dietary iron and release small amounts (1–2 mg/day) to compensate for non-specific losses. Hepatocytes store excess of body iron, which can be mobilized to plasma under iron deficiency. Iron efflux to plasma from macrophages, enterocytes or hepatocytes is negatively regulated by hepcidin, a liver-derived peptide hormone that binds to the iron exporter ferroportin and promotes its degradation (Pantopoulos, 2018—with permission) [18].

Of the 3750 mg of total iron, around 65% of this is incorporated into heme in erythrocytes, 10% within heme of myoglobin of the muscles, 14% in macrophage cells of the reticuloendothelial system (RES), 28% stored within ferritin in hepatocytes and 4% in bone marrow cells [14]. Iron taken up by enterocytes in the gut is stored transiently in small amounts in ferritin until there is demand for iron replenishment elsewhere in the body and then transferrin takes up this iron into the bloodstream, serving as a shuttle/delivery protein chaperone. The transferrin pool is normally only 30% saturated with iron and, therefore, it provides iron-holding capacity to limit amounts of labile non-transferrin-bound iron in circulation. The transferrin iron pool is highly dynamic and represents a flux of around 25 mg iron per day (Pantopoulos, 2018—see Figure 1) [18]. The body has no excretory mechanism for iron and small daily losses of 1–2 mg from skin desquamation and other sources are correspondingly compensated by uptake from the gut.

Hepcidin, a 25-amino acid peptide hormone produced primarily by liver hepatocytes, is the master regulator of iron homeostasis and it negatively regulates iron uptake by inactivating plasma membrane-bound ferroportin on enterocyte and macrophage cell membranes as needed for transfer of intracellular iron to extracellular compartments [19]. This provides tight regulation of iron homeostasis, which is critical for ensuring no excess toxic quantities of iron either intracellularly or extracellularly. Iron dysregulation often presents with elevated amounts of circulating plasma iron including transferrin-bound iron with >50% saturation along with elevated non-transferrin-bound iron (NTBI) that can reach >0.5 µM [20]. It is the labile portion of NTBI that plays a role in the pathogenesis of the various non-microbial diseases we review below, while elevated plasma iron in the form of transferrin-bound iron can also support microbial infection [21].

## 3. Iron Dysregulation Underlying Disease

### 3.1. The Nature of Iron Dysregulation

Iron dysregulation diseases often result from increased amounts of circulating labile reactive plasma iron. This can be evidenced by elevated plasma transferrin-bound iron (TBI) with transferrin saturation above its normal 30%, sometimes reaching 100% saturation [22]. With high saturation of transferrin, some of the plasma iron is not effectively chaperoned and this provides a pool of non-transferrin-bound iron (NTBI) labile reactive iron (LPI). This LPI is also more mobile and capable of entering cells, where it is toxic [23,24].

Excess plasma iron in the form of TBI and NTBI LPI influences two main categories of diseases. Elevated plasma iron, especially in the form of transferrin iron, supports cell-proliferative diseases including infection and cancer. Increased plasma iron present as non-transferrin-bound LPI is part of the pathology of other diseases where excess reactive iron triggers iron-related pathologies. The various diseases we review are summarized in Table 2.

### 3.2. Infections

All pathogenic microorganisms, with the exception of *Borrelia burgdorferi*, the agent of Lyme disease, have absolute and irreplaceable requirements for iron for their growth and replication in our bodies [37]. *B. burgdorferi* can utilize manganese in place of iron [38]. The vertebrate host normally maintains conditions of low iron bioavailability to microbes, especially in extracellular compartments such as plasma, respiratory secretions and tears where infection is often initiated, thus providing a natural nutritional immunity. Transferrin in plasma and lactoferrin in tears and other secretions maintain very low levels of freely available iron [39]. Lactoferrin concentrations can reach >3 mg/mL in tears [40]. Furthermore, when infection is first detected, the body mounts an early iron withdrawal defense response, where extracellular iron concentrations are further reduced by moving this iron to intracellular stores [19]. Successful pathogens deploy a variety of virulence mechanisms to successfully compete for iron, as reviewed elsewhere [21].

#### 3.2.1. Pathogenic Microbes

Iron has now been found to support infection by almost all pathogenic microbes and Table 3 summarizes some important microbial infections in relation to the main host iron sources feeding the infection. Of these, some have cell-wall surface receptors to intercept host iron sources, while others deploy high-affinity siderophores to effectively strip iron from host transferrin or lactoferrin [21].

Interfering with bacterial iron acquisition provides a new therapeutic avenue to fight infection, as discussed later in this review.

#### 3.2.2. Bacterial Sepsis

Sepsis is a life-threatening condition killing around 11 million people worldwide annually [51]. It results from a severely dysregulated host response to inflammation. While sepsis can develop form viral infections such as COVID-19 [51], it most often develops through microbial infection. Bacterial sepsis can develop from serious bacterial infection with the host inflammatory response overstimulated and becoming dysregulated [52], with this often triggered by bacterial cellular components released during infection, including endotoxin [53].

During sepsis, iron metabolism is altered with increased iron uptake into cells and this has been associated with increased iron-driven oxidative injury and cell death [52]. High serum iron levels are also associated with sepsis and poor sepsis outcomes [54]. In fact, transferrin saturation reflecting serum iron availability is strongly correlated to sepsis outcomes, with increased iron availability pronounced in lethally ill patients [54,55]. This aspect, as driven by excess iron, is additional to iron feeding microbial infection, as it relates to host dysregulated inflammation. Thus, the condition of iron dysregulation provides for perfect storm conditions of increased iron availability to support rapidly proliferating microbes on the one hand and increased host damage from the associated dysregulated inflammatory response on the other hand.

Sepsis therapy presents a large unmet medical need with no currently approved therapeutics available for clinical use. However, the underlying iron dysregulation provides a new therapeutic avenue for serious infection/sepsis, as discussed later in this review.

#### 3.2.3. Parasitic Pathogens

Multicellular parasitic organisms also require iron to invade and mount infection in vertebrate hosts [26]. Leishmania chagasi, which causes leishmaniasis, can utilize iron from transferrin, lactoferrin or heme when growing in its promastigote form, taking these up directly without deployment of iron-intercepting siderophores [56]. Trypanosoma brucei employs transferrin surface receptors and Entamoeba histolytica employs lactoferrin receptors for iron acquisition [26]. Plasmodium falciparum, which causes malaria, has also been shown to possess surface receptors for transferrin iron, which is interesting in that it resides within hemoglobin-rich erythrocytes [57]. Addition of iron has been shown to promote infection with Trypanosoma cruzi [58] and iron supplementation has been associated with increased risk of malaria [57]. Parasitic infection, in contrast to most microbial infections, is chronic in nature and has been associated with what is described as the anemia of chronic disease [59]. This also occurs in cancer, as further discussed later, and this chronic anemia is considered the result of the prolonged effort of the host to restrict iron to the invading pathogens or cancer cells. Interestingly, host iron deficiency has been found to be protective against malaria, based on epidemiological studies [57].

#### 3.2.4. Viral Infections

Viruses have no direct requirements for iron, but it is now evident that viral replication in host cells is affected by host iron in the case of both DNA viruses, such as hepatitis B (HBV) and human cytomegalovirus (HCMV), and RNA viruses, such as hepatitis C (HCV) and human immunodeficiency virus (HIV) [27]. It remains somewhat obscure as to whether iron dysregulation predisposes the host to these viral infections, or alternatively, if the viral infection leads to host iron dysregulation. Elevated iron levels are associated with the progression of chronic HBV infection [60] and iron addition has been shown to enhance HCV viral replication in vitro [61]. Transferrin receptor-1 mRNA levels were increased due to HIV infection, leading to an increased iron uptake and higher level of cellular iron [62]. However, it has been suggested that in cirrhotic patients, HBV-related liver injury, but not the HBV infection itself, may be cause changes in serum iron markers [63].

### 3.3. Cancer

The role of iron and its dysregulation in cancer is multi-faceted, as reviewed by Torti et al. (2018) [64]. Stevens et al. (1994) reported on a cohort of 14,000 US National Health and Nutrition survey participants with a major finding that participants with higher transferrin (Tf) iron saturation levels were at higher risk of cancer than participants with lower transferrin Tf saturation levels, a finding supported by subsequent studies [65]. Cancer cells arising from various tissues such as breast or liver have increased need for iron compared to normal cells and produce increased amounts of cell-surface transferrin receptors in response to their increased and continuous iron needs [66]. Targeting transferrin receptors has been proposed for its therapeutic potential [67] as shown using antibodies directed to cancer-cell-surface transferrin receptor TfR1 [68]. This aspect has great appeal as common features to all cancers are possession of TfR1 receptors [68], their altered iron metabolism and increased needs for iron to support their rapid growth [69] and their primary dependence on host transferrin as the source of this iron. Thus, any advances in regard to beneficial iron restriction could apply to various cancers broadly.

Cancer also induces a chronic anemia, as seen in other chronic diseases, and this appears to be a host attempt to restrict cancer growth [64]. Somewhat paradoxically, cancer cells are more susceptible to ferroptosis, a programmed cell death triggered by excess reactive iron [70], suggesting host iron withdrawal defense to restrict cancer growth could also impede cancer cell killing via ferroptosis.

### 3.4. Ferroptotic Cell Death

Ferroptosis is an additional form of regulated cell death (RCD), but unlike other forms such as apoptosis and necrosis, it is caused by an iron-dependent accumulation of lipid peroxides which kill cells, although it shares other common features with the other modes of RCD [70]. ROS produced through the iron-catalyzed Fenton reaction contribute to its initiation [71] and, therefore, iron dysregulation with its excess labile iron is a predisposing factor.

Potential inhibitors of ferroptosis have been investigated including ROS-trapping antioxidants, such as α-tocopherol and ferrostatin-1, and these have shown potential for reducing ferroptotic damage [72].

Excess labile iron driving ferroptosis has also been demonstrated through its suppression by addition of the iron chelator deferoxamine [73]. However, ferric nitriloacetate (NTA) has been shown to promote ferroptosis and renal carcinoma by driving Fenton activity [71]. It is important to note that various iron chelators differ in their abilities to fully coordinate and, therefore, fully chaperone iron [21]. Deferoxamine can fully hexadentate-coordinate iron within a single deferoxamine molecule, while NTA requires two chelator molecules to fully satisfy a single iron atom. Therefore, depending on the prevailing chelator and iron concentrations, incompletely coordinated, Fenton-reactive species can be formed, a problem which appears to underlie the toxicity of some of the currently used medical chelators [21]. This problem of small molecule cell-permeable chelators is compounded in that they can more readily penetrate cells, reaching cellular iron stores and, therefore, mobilize additional labile iron supplies, this in turn exacerbating iron dysregulation.

The excess supply of labile reactive iron observed as a feature of iron dysregulation appears to underpin tissue toxicity with a number of diseases and ferroptosis appears to be at least part of the pathology of these diseases, as further discussed below.

### 3.5. Inflammatory Diseases

While inflammation typically accompanies infection and cancer, other diseases appear to be primarily inflammatory in nature and iron dysregulation and its associated ROS activity are involved in the inflammatory response.

#### 3.5.1. Ocular

Iron dysregulation of the eye has been linked to various eye diseases affecting the cornea and retina, including corneal epithelial disease, corneal endothelial cell dysfunction, retinal pigment epithelial (RPE)-associated eye diseases, glaucoma, diabetic retinopathy (DR), retinal ischemia/reperfusion injury (RIRI), retinoblastoma, retinitis pigmentosa (RP) and age-related cataracts, and common to these, ferroptosis pathology has been implicated [74]. Ferroptotic iron-induced toxicity through ROS damage has been directly linked to corneal diseases such as cataractogenesis, inflammatory retinal diseases such as age-related macular degeneration (AMD) and optic neuropathies [35]. Transferrin mRNA levels are also upregulated in AMD, possibly in response to increased retinal iron loads [75]. This feature provides a potential avenue to new therapeutics, including the use of iron chelators to treat eye diseases that are in urgent need of new therapeutics. 

#### 3.5.2. Lung Fibrosis

Pulmonary iron content is also tightly regulated, as excess iron can catalyze ROS formation, and this has been linked to the pathogenesis of chronic inflammatory lung diseases such as idiopathic pulmonary fibrosis [32]. Iron accumulation was increased in lung sections from patients with IPF, and human lung fibroblasts show greater proliferation and cytokine and extracellular matrix responses when exposed to increased iron levels [76]. These authors provided direct evidence for iron overload affecting the progression of pulmonary fibrosis. They investigated whether changes in iron homeostasis are the cause or a consequence of pulmonary fibrosis and used mouse models of iron overload to show that iron accumulation results in impaired lung function and subsequently worse pulmonary fibrosis upon lung injury by bleomycin [76]. Lung fibrosis is a progressive irreversible disease, as fibrotic lung tissue does not repair/remodel, making anti-fibrotic agents an urgent need. Overcoming iron dysregulation to reduce iron-driven fibrosis is a new potential avenue for therapy. Iron chelation therapeutics have potential for use in slowing or stopping pulmonary fibrosis.

#### 3.5.3. Kidney

Increased kidney proximal tubule (PT) cell cytosolic non-transferrin Tf-bound, i.e., labile iron has been shown to induce the generation of ROS in PT cells and this could contribute to the progression of proteinuric chronic kidney diseases [34]. Recent clinical studies using anti-oxidative drugs that serve to reduce labile iron suggest that chelation of iron in the kidney has beneficial effects on the course of chronic kidney disease [77]. In nephritic syndromes associated with damage to the glomerular filter, urinary Tf concentration dramatically increases and may even lead to hypo-transferrinemia, iron loss and microcytic anemia [78].

Nephritic kidney damage with urinary transferrin excretion is also part of the pathology associated with autoimmune diseases including lupus erythematosus [79] and rheumatoid arthritis [80], as further discussed further below.

### 3.6. Diabetes

High dietary iron and dysregulated iron metabolism are risk factors for type 2 diabetes mellitus (T2DM), affecting most of its features of decreased insulin secretion, insulin resistance and increased hepatic gluconeogenesis [29]. Dysregulated iron metabolism with increased serum levels of ferritin have been found in newly diagnosed type 2 diabetes but not in individuals with pre-diabetes [81]. Oxidative stress from ROS is now known to be a major underlying mediator of diabetic complications [82]. Iron and ferroptosis have been shown to participate in pancreatic beta cell death [83]. In model studies using T2DM, mice insulin secretion was worsened by ferroptosis-inducing compounds. However, quercetin (a natural iron chelator), ferroptosis inhibitor ferrostatin-1 and iron-chelating deferoxamine, each rescued cell viability when cells were challenged with high glucose [84]. These studies support the potential for use of iron-chelating therapeutics in the treatment of T2DM.

### 3.7. Cardiovascular

Ferroptosis driven by iron dysregulation has now been implicated in several cardiovascular disease conditions including cardiomyopathy, atherosclerotic disease and myocardial ischemia/perfusion injury [30,85]. Inhibition of ferroptosis by ferrostatin-1 improved cardiac function and reduced mortality in a doxorubicin-induced mouse model of cardiomyopathy, which was associated with the release of free cellular iron caused by HO-1 upregulation [86]. However, as pointed out by Li and Zhang (2021), the mechanisms of ferroptosis in heart and vasculature disease remain elusive [30].

### 3.8. Autoimmune

The role of iron regulation in immune-related diseases was recently reviewed by Cronin et al. (2019) [87]. Substantial evidence has now linked iron dysregulation to the pathogenesis of lupus erythematosus [88]. Importantly, the common serious complication, lupus nephritis, has been linked to renal iron accumulation [79] and ferroptosis kidney cell damage has been described as an important feature of its pathology [88].

### 3.9. Neurological

Many neurological conditions have been shown to coincide with altered bodily distribution of various transition series biometals, especially in the case of iron [89]. Iron dysregulation with increased labile plasma iron supply and resulting increased ROS, creating oxidative stress and damage on neurological tissues, has now been linked to Friedreich’s ataxia, Alzheimer’s disease (AD), Parkinson’s disease, Multiple Sclerosis (MS) and Amyotrophic Lateral Sclerosis (ALS) [31]. Increased ferroptosis with corresponding destruction of neurological cells has been described in Parkinson’s disease and MS [90] as well as for Alzheimer’s disease [91].

For example, Parkinson’s disease (PD) is characterized by progressive motor impairment attributed to progressive loss of dopaminergic neurons in the substantia nigra (SN) pars compacta. In addition to an accumulation of iron, there is also an increased production of reactive oxygen/nitrogen species (ROS/RNS) and inflammatory markers seen in this pathology [92]. In addition, abnormal iron increases are commonly detected in AD patients, although controversy continues regarding iron’s association with AD plaques [91]. It now appears that a common feature of iron dysregulation underlying neurological diseases is iron-driven ferroptosis neurological cell death [93].

### 3.10. Iron Overload

While body iron overload can follow repeated blood transfusions, as seen in patients with Thalassemia, i.e., because the body lacks an excretory pathway for excess iron, congenital disorders of iron overload such as hemochromatosis represent iron overload disorders caused by dysregulated iron homeostasis. In hemochromatosis, disruption of the hepcidin pathway due to mutations in genes encoding auxiliary factors in iron signaling to hepcidin result in insufficient hepcidin responses to iron intake or to high body iron stores. This causes loss of hepcidin-mediated feedback inhibition in dietary iron absorption and consequently unregulated uptake of dietary iron, elevated transferrin iron saturation and appearance of labile reactive non-transferrin-bound iron [18].

### 3.11. Cirrhosis

Non-alcoholic fatty liver disease (NAFLD) is the most common chronic liver disease worldwide, beginning with the presence of >5% excessive lipid accumulation in the liver, and typically developing into non-alcoholic steatohepatitis, fibrosis, cirrhosis and often hepatocellular carcinoma. Excess free reactive iron and its associated ROS-mediated tissue damage have been positively associated with severity of NAFLD [36].

### 3.12. Anemia of Chronic Infection and Inflammation

The anemia of inflammation (AI), often referred to as the anemia of chronic disease (ACD), is a secondary anemia that often develops slowly as a result of chronic inflammation during chronic infection (especially parasitic), cancer and with at least some of the other inflammatory diseases we have reviewed, such as diabetes and autoimmune diseases [59,94,95]. Overall, it appears to be a host defensive mechanism. Interestingly, a short-term hypoferremia response is often seen early in the acute stages of infection and this has also been shown to be triggered by inflammatory mediators such as ILK-6 and demonstrated to be a relatively short-term active mechanism for restricting iron supply to growing invaders [21]. The diagnostic challenge in AI/ACD is the identification of patients with concomitant true iron deficiency because they need specific evaluation for the source of blood loss and iron-targeted management strategies [Weiss et al. 2019].

## 4. Therapeutic Options

### 4.1. Iron Restriction

Higher heme iron intake and increased body iron stores were significantly associated with a greater risk of type 2 diabetes, as shown in a meta-analysis of 11 prospective studies [96]. Interestingly, blood-letting to reduce overall body iron stores has shown potential benefit in the treatment of diabetes [97].

In principle, restriction of iron uptake would also lower amounts of deleterious liable reactive iron. However, withholding (or supplying) iron has remained controversial in medicine over the years due to the delicate balance required for iron homeostasis and the consequences of triggering anemia. While supply of dietary iron would be safer, administration of parenteral iron to treat anemia, especially the anemia of chronic disease, has serious implications. Given the roles of dysregulated iron in infection and other diseases, this requires careful consideration and likely should be avoided if possible.

### 4.2. Iron Chelators

Iron chelators that bind and can sequester excess available labile reactive iron have shown potential for the therapy of several diseases associated with iron dysregulation. However, medical chelators to date have been developed to treat hematological conditions associated with high excess body iron stores (e.g., transfusional iron overload in Thalassemia patients) but were not developed as iron dysregulation therapeutics. We have provided a number of important potential attributes for ideal iron dysregulation chelator therapeutics, as summarized in Table 4.

A number of clinical trials or natural products with iron-chelating properties, such as curcumin and polyphenolics, have shown promise for lowering excess iron [105] but none have been approved for clinical use.

### 4.3. Hepcidin and Agonists

Hepcidin mimetics, stimulators of its production and ferroportin inhibitors have seen early clinical stage testing as to their safety and potential efficacy [106]. The ferroportin inhibitor VIT-2763 has advanced to phase II trials and it has shown both low toxicity and good potential for lowering serum iron levels [107] Other approaches of using hepcidin mimetics including PTG-300 (rusfertide) have shown promising phase III results. As of yet, none of these have received regulatory approval for ongoing clinical use [108].

## 5. Future Needs and Conclusions

We identified a number of gaps in our current understanding of iron dysregulation in the pathology of diseases. A better understanding of the role of Fe dysregulation mechanisms for various diseases, especially related to cause or effect, is needed. There is a particular need for further understanding of anemia of chronic disease. Possible targeted ferroptosis induction in cancer can be considered once its mechanisms are elucidated.

A number of conclusions can be drawn at this stage. Therapeutics affecting iron dysregulation and lowering excess levels of labile reactive ion should be readily applicable to treating infection, including sepsis, and should also apply to cancer and other non-proliferative diseases from iron dysregulation. Improved chelators have immediate appeal as the broadest and preferred therapeutic approach.

Hepcidin mimetics and agonists have demonstrated potential and longer-term potential.

## Figures and Tables

**Table 1:** Iron-dependent enzymes.

Enzyme	Function	Reference
Cytochrome oxidase EC 7.1.1.9	Energy production	Williams, 1987 [2]
Citrate aconitase EC 4.2.1.3	TCA cycle	Beinert et al., 1996 [3]
Ribonucleotide reductase EC 2.7.7.56	DNA synthesis	Jordan and Reichard, 1998 [4]
DNA polymerase EC 2.7.7.7	DNA replication and repair	Zhang et al., 2014 [5]
Oxoglutarate oxygenase EC 1.13.12.19	Lipid biosynthesis	McDonough et al., 2010 [6]
Superoxide dismutase EC 1.15.1.1	Superoxide radical detoxification	Parker and Blake, 1988 [7]
Catalase EC 1.11.1.21	Peroxide detoxification	Alfonso-Prieto et al., 2009 [8]

**Table 2:** Diseases with iron dysregulation.

Disease Category	Disease	Example	Reference
Proliferative Cell Replication	Microbial Infection	Staphylococcal, Candidiasis	Holbein et al., 2021 [21]
	Bacterial Sepsis	*Pseudomonas aeruginosa*, *Neisseria meningitidis*	Bullen et al., 2000 [25]
	Parasitic Infection	Chagas disease, Malaria	Mach and Sutak, 2020 [26]
	Viral Infection	HIV, HBV, HCV, HCMV	Schmidt, 2020 [27]
	Cancer	Pancreatic, Liver, Lung	Torti et al., 2018 [28]
Iron-Mediated Pathology	Diabetes	Type 2 Diabetes	Harrison et al., 2023 [29]
	Cardiovascular	Cardiomyopathy	Li and Zhang, 2021 [30]
	Neurological	Alzheimer’s, ALS, MS, Parkinson’s	David et al., 2022 [31]
	Lung Fibrosis	Idiopathic Pulmonary Fibrosis	Ogger and Byme, 2020 [32]
	Autoimmune	Rheumatoid Arthritis, Lupus Erythematosus	Baker and Ghio, 2009 [33]
	Kidney	Fanconi Syndrome	Smith and Thévenod, 2009 [34]
	Retinal	Age-Related Macular degeneration	Loh et al., 2009 [35]
	Iron Overload	Hemochromatosis	Pantopoulos, 2018 [18]
	Cirrhosis	Non-Alcoholic Fatty Cirrhosis	Chen, 2022 [36]

**Table 3:** Iron sources for pathogenic microbes.

Microbial Pathogen	Host Iron Source	Reference
*Neisseria meningitidis*	Transferrin	Holbein, 1981 [41]
*Pseudomonas aeruginosa*	Transferrin, Lactoferrin	Xiao and Kisaalita, 1997 [42]
*Acinetobacter baumannii*	Transferrin ^1^, Lactoferrin ^1^, Heme ^2^	^1^ Yamamoto et al., 1999 [43]; ^2^ Giardina et al., 2019 [44]
*Campylobacter jejuni*	Transferrin, Lactoferrin	Miller et al., 2008 [45]
*Staphylococcus aureus*	Transferrin ^3^, Heme ^4^	^3^ Modun et al., 1998 [46]; ^4^ Skaar and Schneewind, 2004 [47]
*Mycobacterium tuberculosis*	Transferrin	Clemens et al., 1996 [48]
*Candida albicans*	Transferrin	Knight et al., 2005 [49]
*Aspergillus fumigatus*	Transferrin	Hissen et al., 2004 [50]

**Table 4:** Iron chelators.

Chelator	DFO Deferoxamine	DEF Deferasirox	DFP Deferiprone	DIBI Hydroxypyridinone
MW (Da)	561	373	139	9000 avg. polymeric [98]
Fe(III): chelator binding complex	1:1	1:2	1:3	3:1 [98]
Fe binding constant log K	30.6 [99]	36.5 [99]	36.7	41.0 [98]
Hydrophobicity/hydrophilicity log P	−3 [99]	4.3 [99]	−0.8 [99]	−1.87 ^a^
FDA Approval	1968	2005	2011	In Development
Treatment Indication	Transfusional iron overload [100]	Transfusional iron overload [100]	Transfusional iron overload [100]	Anti-infective [101] Anti-inflammatory [102]
Serious limitations and toxicities	Promotes infections [21]	Toxicity, Renal failure [103,104]	Toxicity, Agranulocytosis [104]	None to date from oral and systemic animal testing ^b^
Promotes microbial pathogen growth	Very Serious	Potentially Serious	Potentially Serious	No [21]

^a^ Holbein, unpublished CRO report: JBL-DMPK-0062-0016, ^b^ Holbein, unpublished CRO reports: SP-JBL/NG-RIVR-142 and JBL/NG-ROR-052.
